# Adult-Type Rhabdomyoma of the Omohyoid Muscle

**DOI:** 10.1155/2019/4706582

**Published:** 2019-07-30

**Authors:** Brian H. Cameron, Kyle Hannabass, Anuradha Kanungo, Dinesh K. Chhetri

**Affiliations:** ^1^Keck School of Medicine of USC, Los Angeles, CA, USA; ^2^Department of Head and Neck Surgery, David Geffen School of Medicine, University of California Los Angeles, Los Angeles, CA, USA; ^3^Department of Pathology, Olive View-UCLA Medical Center, Los Angeles, CA, USA

## Abstract

Rhabdomyomas are benign tumors composed of mesenchymal tissue and having a histologic appearance similar to skeletal muscle. Extracardiac rhabdomyomas are rare, and the majority of the adult subtype occur in the head and neck (H&N) region. Diagnosis can be challenging due to fine-needle aspiration (FNA) and core needle biopsy being suspicious for sampling error from surrounding muscle or concerning for rhabdomyosarcoma. We present a case of a slowly enlarging left neck mass in the strap musculature of a 45-year-old Hispanic male. Multiple FNA and core biopsies failed to establish a diagnosis, and excisional biopsy was pursued revealing a hypertrophied left inferior belly of the omohyoid muscle. Histological analysis was diagnostic of an adult-type extracardiac rhabdomyoma, with complete surgical excision being the gold standard treatment for these tumors. The patient had an uneventful recovery. Skeletal muscle tumors of the H&N are uncommon, and benign extracardiac rhabdomyoma must be considered in the differential diagnosis to prevent unnecessarily aggressive intervention and appropriate patient counseling before and after surgery.

## 1. Introduction

Rhabdomyomas are benign masses of mesenchymal tissue arising from skeletal muscle. They are extremely rare tumors, are less common than malignant rhabdomyosarcomas, and account for 2% of skeletal muscle tumors.

Rhabdomyomas are either cardiac or extracardiac. Extracardiac rhabdomyomas are less common and were first described by Pendl in 1897. They are slow-growing lesions that consist of three main types: fetal, adult, and genital. A fourth type, rhabdomyomatous mesenchymal hamartoma, is occasionally mentioned. The fetal type more commonly affects children and is often found in the head and neck (H&N) region. This type is further divided into classic and juvenile subtypes. The adult type is commonly found in elderly individuals and occurs in a 3 : 1 male to female ratio. Adult rhabdomyomas can present as unifocal or multifocal lesions. The majority of adult rhabdomyomas (93%) occur in the H&N region: pharynx, larynx, oral cavity, and soft tissue of the neck [1]. The genital type is found commonly in younger women and presents as a polypoid mass on the vagina/vulva. It should be noted that fetal, juvenile, and adult subtypes are differentiated by their histologic characteristics and not by the age of onset [2].

While rhabdomyomas are most often present in the soft tissues of the H&N region, intramuscular occurrence in the muscles of the neck is extremely rare, with fewer than five published cases in English journals. We present an illustrative case of an adult-type rhabdomyoma occurring in the omohyoid muscle.

## 2. Case Report

This case report was exempted from review by the University of California, Los Angeles, Institutional Review Board (IRB).

**Figure 1:**
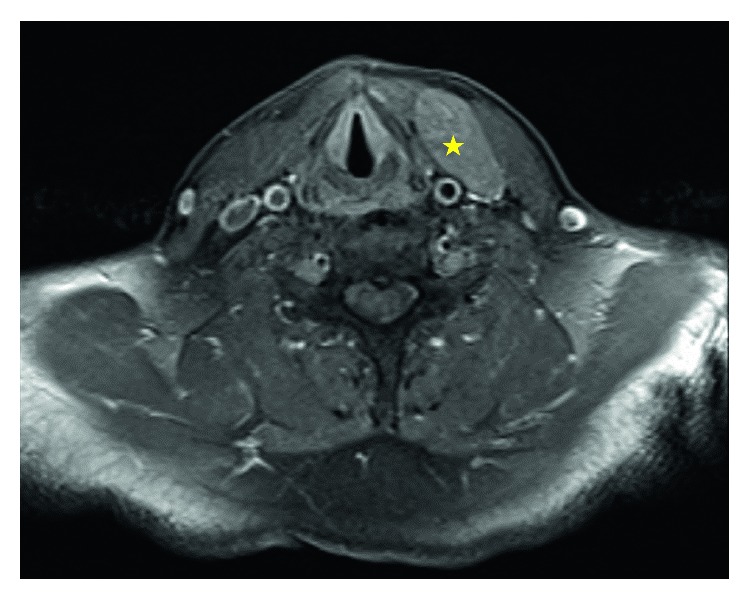
MRI T1 FatSat axial showing lesion (star) within left omohyoid muscle.

A 45-year-old diabetic male presented to the ENT clinic with a 1.5-week history of left neck tenderness and swelling. He denied fever, dysphonia, or dysphagia. He had no history of malignancy or other H&N masses. Physical exam revealed left neck fullness over the sternocleidomastoid muscle. Computed tomography (CT) and magnetic resonance imaging (MRI) of the neck were performed. CT showed an isodense enlargement of the left omohyoid muscle with the greatest dimension of 1.6 cm. MRI showed a 5 × 2 × 4 cm ovoid mass, T1 isointense to muscle, and T2 very slightly hyperintense to muscle, embedded within the left strap musculature (Figure 1). Radiologist described that the mass “may represent a nerve sheath tumor such as a schwannoma versus a possible sarcoma.”

**Figure 2:**
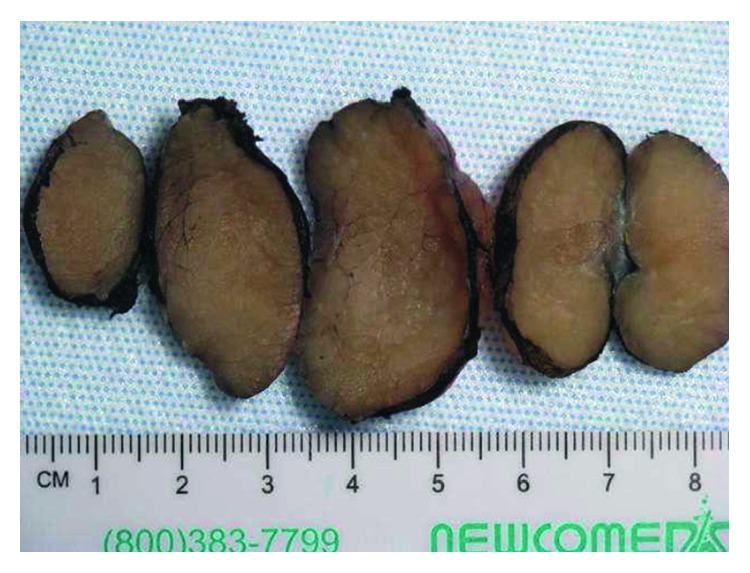
Gross coarsely lobulated cut surface of excised rhabdomyoma. Note: mass appears discolored due to formalin fixation.

Ultrasound-guided fine-needle aspiration and core needle biopsy revealed benign skeletal muscle. A repeat biopsy was performed but only showed skeletal muscle with chronic inflammation. Since the mass was enlarging but lacked a definitive diagnosis, it was excised (Figure 2). Intraoperative findings included ovoid enlargement of the superior belly of the left omohyoid, which was resected in its entirety and sent as a permanent specimen to pathology.

**Figure 3:**
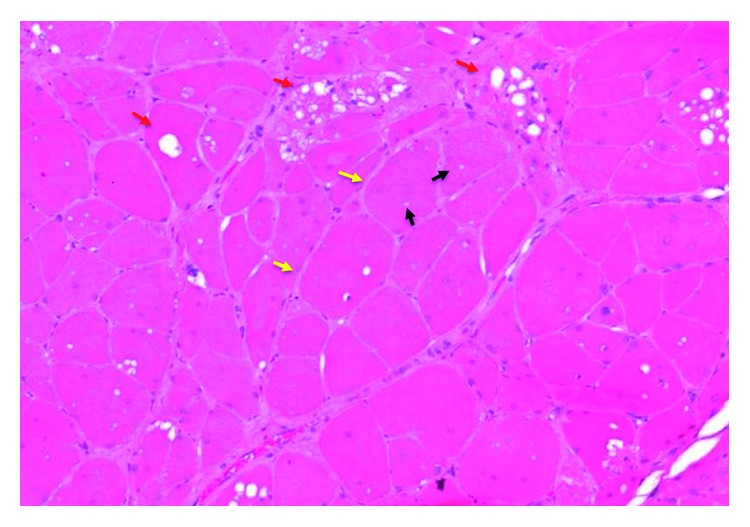
10x magnification of H&E stained histology specimen of rhabdomyoma shows tightly packed, variously sized, round or polygonal cells separated from one another by thin fibrous septa and small vascular channels (yellow arrows). The cells have eosinophilic, striated cytoplasm with occasional intracytoplasmic vesicles (black arrows), vacuoles (red arrows), peripherally placed vesicular nuclei, and one or two prominent nucleoli. Mitotic figures and evidence of necrosis were absent.

Pathologic diagnosis showed skeletal muscle with rare cytoplasmic vacuoles and focal interstitial chronic inflammation consistent with extracardiac adult-type rhabdomyoma (Figure 3). Margins were negative. The patient presented one week after surgery with no complications and a well-healing wound. On follow-up, he remained free of recurrence.

## 3. Discussion

Adult-type extracardiac rhabdomyomas are typically painless, having signs and symptoms related to their size and location. Though not seen in this case, patients have reported feelings of dysphonia, dysphagia, or respiratory distress.

As in this case, diagnosing an extracardiac rhabdomyoma can be difficult, as imaging can be inconclusive. These lesions typically are submucosal and radiologically are not seen to invade adjacent tissue (indicating benign lesion). Their borders, however, can be indistinct, similar to malignant tissues [3]. In addition, FNA and core needle biopsy can show skeletal muscle suggestive of sampling error or sarcoma.

Definitive diagnosis is commonly made after excision and histopathologic examination. Histology commonly shows densely packed polygonal cells with eosinophilic granular cytoplasm, prominent nucleoli, cross-striations, and spider cells (Figure 3) with some cells showing extensive vacuolization. These lesions do not demonstrate any necrosis or mitotic figures. These lesions typically do not express common markers of cellular proliferation such as Ki-67 or proliferating cell nuclear antigen (PCNA), leading many to consider these lesions as hamartomas instead of true neoplasms [4].

**Figure 4:**
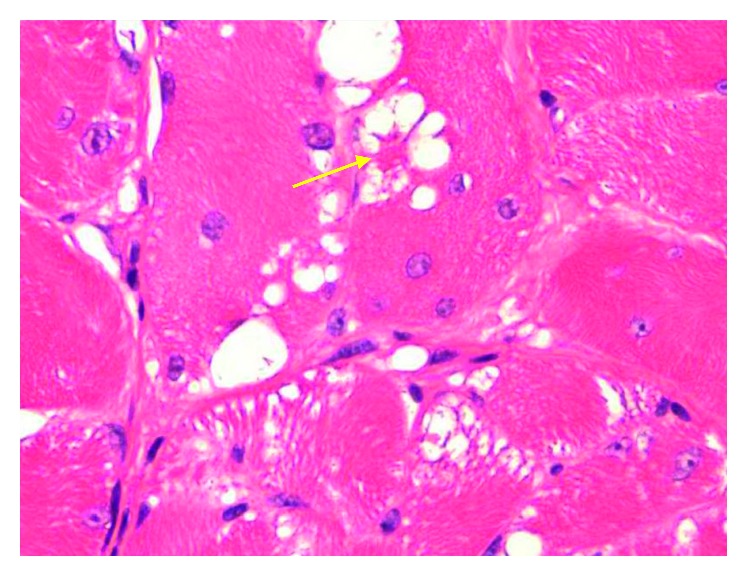
40x magnification of H&E stained histology specimen of rhabdomyoma shows tightly packed polygonal cells separated by thin fibrous septa, vascular channels, striated cytoplasm, and spider cells (arrow), characterized by a central acidophilic mass connected to a condensed rim of cytoplasm at the periphery by thin strands of cytoplasm. Mitotic figures and evidence of necrosis are absent.

Historically, these lesions were often confused with granular cell tumors, as they also commonly occur in the H&N region. Granular cell tumors, however, are composed of Schwann cells and thus are immunoreactive for the S100 antibody and lack the extensive vacuolization (spider cells) and cross-striations seen in rhabdomyomas (Figure 4). Hibernomas should also be considered when suspecting a rhabdomyoma as these lesions do have extensive vacuolization, but these masses are more commonly interscapular and are characterized by frequent cytoplasmic lipid droplets [5].

Surgical excision is the standard treatment of extracardiac rhabdomyomas. While these masses show no malignant potential, complete excision of these lesions is important, as recurrence is seen in up to 42% of cases and is commonly due to incomplete excision or the presence of multifocal lesions [6].
